# Social jetlag and quality of life among nursing students during the COVID-19 pandemic: a cross-sectional study

**DOI:** 10.1186/s12912-023-01223-x

**Published:** 2023-03-03

**Authors:** Sun Joo Jang, Haeyoung Lee

**Affiliations:** Red-Cross College of Nursing, Chung-Ang University, 84 Heukseok-ro, Dongjak-gu, 06974 Seoul, Republic of Korea

**Keywords:** COVID-19 pandemic, Depression, Nursing students, Quality of life, Social jetlag

## Abstract

**Background:**

Amid drastic changes in the educational environment and continued substitution of in-person learning with online learning owing to the COVID-19 pandemic, it is important to analyze the predictors of quality of life among nursing students to devise strategies to enhance their quality of life. This study aimed to identify the predictors of nursing students’ quality of life during the COVID-19 pandemic, focusing on social jetlag.

**Methods:**

In this cross-sectional study, data were collected from 198 Korean nursing students in 2021, using an online survey. Chronotype, social jetlag, depression symptoms, and quality of life were assessed using the Korean version of the Morningness-Eveningness Questionnaire, Munich Chronotype Questionnaire, Center for Epidemiological Studies Depression Scale, and World Health Organization Quality of Life Scale abbreviated version, respectively. Multiple regression analyses were performed to identify the predictors of quality of life.

**Results:**

Factors affecting participants’ quality of life were age (*β* = − 0.19, *p* = .003), subjective health status (*β* = 0.21, *p* = .001), social jetlag (*β* = − 0.17, *p* = .013), and depression symptoms (*β* = − 0.33, *p* < .001). These variables accounted for 27.8% of the variance in quality of life.

**Conclusion:**

As the COVID-19 pandemic continues, the social jetlag of nursing students has decreased compared to before the pandemic. Nevertheless, the results showed that mental health issues such as depression affect their quality of life. Therefore, it is necessary to devise strategies to support students’ ability to adapt to the rapidly changing educational environment and promote their mental and physical health.

## Background

The coronavirus disease 2019 (COVID-19) pandemic has resulted in dramatic changes in people’s daily lives, including systematic changes in the traditional teaching approaches [1]. Since the COVID-19 outbreak, colleges worldwide have completely or partially shifted to an online learning system [2–5]. Nursing is no exception; as communities, hospitals, and colleges shifted from traditional, in-person nursing education, alternative educational methods such as online learning were applied and their effectiveness was evaluated in South Korea [6, 7]. However, only 20.4% of medical and nursing students believed that e-learning can replace conventional teaching [8], and many health professional students (98.2%) experienced moderate-to-high level stress [9]. Many students experienced difficulties in effectively engaging in online learning amid dramatic changes in the educational system owing to the pandemic [10, 11]. They experienced lowered adjustment to and satisfaction with college life [12] and developed concerns and anxiety about their academic performance and long-term employment [13]. With students’ academic stress being higher than ever, there is a pressing need for governmental effort and measures to address it [14].

Changes in school and daily life aggravate students’ mental health problems [15] and diminish life satisfaction and health-related quality of life (HRQOL) [16, 17]. Restrictions on students’ activities owing to the COVID-19 pandemic are significantly associated with impaired quality of life (QOL) [18], which in turn reduces academic performance [19]. A study that investigated factors associated with QOL in college students during the COVID-19 pandemic reported that their psychological and social relationship QOL had relatively decreased [20]. This was linked to a high incidence of depression, anxiety, and stress in college students [20]. Moreover, family, friends, and other forms of significant social support have been identified as the predictors of increased psychological QOL [20], suggesting that social support for college students is crucial during lockdowns.

College students are vulnerable to mental health problems and display a high level of stress as they transition from adolescence to adulthood and sustain academic pressure, which can negatively impact academic performance, social functioning, and QOL [21–23]. During the COVID-19 pandemic, college students experienced psychological distress, such as stress, depression, anxiety, and sleep problems [24, 25]. This has been associated with COVID-19-related economic problems, impacts on daily life, and concerns about academic delays [24]. A study reported that nursing students exhibit lower general health, a higher level of psychological distress, and lower overall QOL since the outbreak of COVID-19 compared to before [26]. Even in normal circumstances, nursing students experience a higher level of stress compared to other health professional students [27]. The key causes of such stress include test-taking anxiety [28], heavy didactic and clinical workloads [29], feeling unprepared for practice, and fearing mistakes [30]. Considering that resilience, having online experience, and being well prepared for online learning have been significant predictors of QOL in nursing students during the COVID-19 pandemic [31], it is highly likely that changes in the educational system owing to the pandemic and consequent stress experienced as students adapt to the changes have an impact on nursing students’ mental health and QOL. As it is unknown when COVID-19 will be eradicated, it is important to support nursing students to adjust to the changed educational system and adapt to restrictions in the practicum courses to boost their academic performance and promote their mental health and QOL.

The COVID-19 pandemic has some positive effects; for instance, nursing students reported longer sleep during school closures [32]. Considering the reports that delayed school start time increases students’ sleep duration, which has a positive impact on the health of adolescents [33, 34], changes in students’ sleep because of school closure during the pandemic could be interpreted in the same context [32]. A study of young Japanese adults found that their total sleep time and degree of social jetlag (SJL) had improved since the outbreak of COVID-19, and that students had a significantly delayed sleep phase than workers [35]. However, there was also a report that most college students (73.3%) had poor quality of sleep during the COVID-19 lockdown [36], highlighting the need to examine the impact of changes in sleep owing to the pandemic and consequent SJL on health and QOL in college students.

SJL refers to a misalignment between an individual’s circadian rhythm—an endogenous sleep-wake cycle—and social rhythm; that is, an asynchrony of an individual’s circadian preference and social time [37]. SJL has a negative impact on physical and mental health, with those with greater SJL being more likely to be depressed [38] and having worse QOL than their counterparts [39]. College students are often active until late night and go to bed late owing to their age-dependent delayed endogenous circadian clock and late chronotype; consequently, inadequate sleep duration has negative effects on the mental health [40], HRQOL [41, 42], and academic performance [43, 44] of young populations. Therefore, this study examined the level of SJL experienced by nursing students amid COVID-19-induced changes in daily living and educational environments and investigated the effects of SJL and physical and mental health-related factors on their QOL.

## Methods

### Study design

This study utilized a cross-sectional design.

### Participants and data collection

This study was conducted at six universities in South Korea. The inclusion criteria were (1) current nursing students in university, (2) completion of the 2021 spring semester during the third wave of the COVID-19 pandemic, and (3) voluntarily providing informed consent. The exclusion criteria were (1) not being enrolled in school and (2) having a physical or mental disease.

The minimum sample size for multiple regression analyses was determined using G*power 3.1.9.2. With a significance level of 0.05, power of 0.95, 11 predictor variables, and an effect size (f^2^) of 0.15 (medium), the minimum sample size was 178. Considering a 10% withdrawal rate, a target of 198 participants was set. Data were collected using an online survey from September to December 2021. As of October 1, 2021, when data collection was in progress, the COVID-19 estimates for South Korea were 2,451 new confirmed cases, 313,773 total confirmed cases, and 2,497 cumulative deaths (0.80% fatality rate); the stringency index was 61.90. Information and a link to the study questionnaire were posted on the official college homepage and social media account (i.e., student council or club) for nursing students at universities. The survey link served as an open call; students who were enrolled at the college during the survey period and wished to participate could do so, provided they met all the inclusion criteria. A warning was displayed to the participants not to respond more than once. In total, 198 participants met the inclusion criteria; therefore, all responses were analyzed.

### Ethical considerations

This study was approved by the institutional review board at the authors’ affiliated C University (no. 1041078-202101-HRSB-004-01, approved on March 8, 2021). Electronic informed consent was obtained from those students who consented to participate in the study. They were informed that they could withdraw from the study at any time by contacting the authors at the numbers provided.

### Instruments

#### General and health-related characteristics

Participants’ general characteristics (age, gender, school year, grade point average [GPA], living arrangement, and socioeconomic status) and health-related characteristics (subjective health status, smoking, drinking, height, and weight) were identified using a self-report questionnaire.

#### Circadian parameters

Chronotype was measured using the Korean version [45] of the Morningness-Eveningness Questionnaire (MEQ-K) [46]. The MEQ-K comprises 19 items, with a total scoring range of 16–86 points (11 items [scoring range 1–4 points]; 2 items [scored 0, 2, 4, and 6]; 1 item [scored 0, 2, 3, and 5]; 5 items [scoring range 1–5 points); a higher score indicates a more extreme morning type [46]. Based on the criteria of total scores, chronotype was categorized into three groups: morning chronotype (59–86), neither chronotype (42–58), and evening chronotype (16–41) [47]. The reliability (Cronbach’s α) of the MEQ was 0.82 at the time of development [46], 0.77 for MEQ-K [45], and 0.64 in this study.

The SJL was measured using the Munich Chronotype Questionnaire (MCTQ) [48]. The MCTQ comprises 14 items about bedtime, sleep onset, sleep latency, time of awakening, time to get up, use of an alarm, and outdoor activity time, to assess weekday and weekend sleep-wake cycles. Cheng and Hang [49] established the reliability of the MCTQ by confirming that the sleep-wake patterns measured using the scale are closely linked to actigraphy results. SJL was calculated based on the sleep-corrected SJL formula by Jankowski [50].

Daily light exposure was calculated based on the response on the MCTQ about the time spent outdoors without a roof during the daytime on weekdays and holidays. Sleep duration was calculated as the time from sleep onset to time of awakening based on the response on the MCTQ.

#### Depression symptoms

The Korean version of the Center for Epidemiological Studies Depression Scale (CES-D) [51] was used to measure depression symptoms in participants. The 20-item CES-D measures depression symptoms in the past week using a four-point Likert scale from 0 to 3 (scores range from 0 to 60), and a higher score indicates more severe depression. Cronbach’s *α* was 0.91 and 0.83 in the Korean version of the CES-D [51] and in this study, respectively.

#### QOL

The Korean version of the World Health Organization QOL Scale abbreviated version [52] was used to measure QOL. This 26-item tool comprises four subdomains (physical health, psychological health, social relationships, and environment) and measures QOL in the past two weeks using a five-point Likert scale (scores range from 26 to 130). Cronbach’s *α* of the Korean version was 0.90 previously [52] and 0.93 in this study.

### Data analysis

Data were analyzed using SPSS Statistics 26.0 software (IBM, Armonk, NY, USA). Data normality was tested using the Kolmogorov–Smirnov test. Harman’s single-factor test was used to identify common method bias [53]. Differences in QOL according to participants’ characteristics were analyzed with *t*-tests and analyses of variance. The relationships among general and health-related characteristics, circadian parameters, depression symptoms, and QOL were analyzed using Pearson’s correlation coefficients. The predictors of nursing students’ QOL were analyzed using multiple regression analyses. Further, Kolmogorov-Smirnov’s normality and Breusch-Pagan’s homoscedasticity tests were performed.

## Results

### General and health-related characteristics

The median age of the study population (N = 198) was 22.00 years. The majority (87.9%) of the students were female, and 38.9% were fourth-year students. Approximately half (55.1%) of the students lived with others, and 52.0% were of middle socioeconomic status. Furthermore, 44.9% rated themselves as healthy. Regarding smoking and drinking status, 92.9% and 57.6% were non-smokers and non-drinkers, respectively, and 61.6% had a normal body mass index (BMI) (Table 1).

**Table 1:** Participants’ general and health-related characteristics (*N* = 198)

Characteristic	Category	*n* (%)	Median (interquartile range)	*p* ^*b*^
Age (years)			22.00 (21.00 ~ 23.00)	< 0.001
Gender	Male	24 (12.1)		
	Female	174 (87.9)		
School year	First	12 (6.6)		
	Second	55 (27.8)		
	Third	53 (26.8)		
	Fourth	77 (38.9)		
Grade average point			3.00 (2.00 ~ 4.00)	< 0.001
Living arrangement	Alone	89 (44.9)		
	With others	109 (55.1)		
Socioeconomic status	Low	41 (20.7)		
	Middle	103 (52.0)		
	High	54 (27.3)		
Subjective health status	Unhealthy	28 (14.1)		
	Healthy	170 (85.9)		
Smoking	Yes	14 (7.1)		
	No	184 (92.9)		
Alcohol	Yes	84 (42.4)		
	No	114 (57.6)		
Body mass index^a^	Underweight	34 (17.2)	20.79 (19.12 ~ 22.61)	< 0.001
	Normal	122 (61.6)		
	Overweight	19 (9.6)		
	Obese	23 (11.6)		

^a^Per the World Health Organization’s classifications, suggested and revised for the Asia-Pacific region by Hoque et al. [54].

^b^Kolmogorov-Smirnov test results.

### Circadian parameters, depression symptoms, and QOL of participants

Majority of the participants (71.2%) were of neither chronotype. The median SJL was 1 h and 19 min. The mean sleep duration was 7 h and 9 min (standard deviation: 1 h 30 min) and median daily light exposure was 1 h and 24 min. The median depression symptoms and mean QOL scores were 11.00 and 94.36 (standard deviation: 18.02), respectively (Table 2).

**Table 2:** Circadian parameters, depression symptoms, and quality of life

Variable	Category	*n* (%)	*M* (*SD*)	Median (interquartile range)	*p* ^a^
	Evening	50 (25.2)	46.18 (7.06)		0.141
Chronotype	Neither	141 (71.2)			
	Morning	7 (3.5)			
Social jetlag				1 h 5 min (30 min ~ 1 h 59 min)	0.018
Sleep duration			7 h 9 min (1 h 30 min)		0.915
Light exposure				1 h 24 min (51 min ~ 2 h 23 min)	0.001
Depression symptoms				11.00 (8.00 ~ 17.00)	< 0.001
Quality of life			94.36 (18.02)		0.750

M: mean, SD: standard deviation

^a^Kolmogorov-Smirnov test results

### Differences in QOL according to participants’ characteristics

Table 3 shows the differences in QOL according to participants’ general and health-related characteristics. QOL did not statistically significantly differ according to gender, school year, living arrangement, socioeconomic status, smoking and drinking status, chronotype, or BMI. Those with a good subjective health status had a higher QOL than those with a poor subjective health status.

**Table 3:** Differences in quality of life according to participants’ characteristics

Characteristic	Category	*M* (*SD*)	*t*/*F* (*p*)
School year	First-year	97.77 (17.88)	0.34 (0.797)
	Second-year	94.84 (17.60)	
	Third-year	92.60 (17.17)	
	Fourth-year	94.66 (19.10)	
Living arrangement	Alone	93.88 (17.85)	0.34 (0.732)
	With others	94.76 (18.22)	
Socioeconomic status	Low	91.37 (17.48)	1.37 (0.257)
	Middle	93.96 (17.50)	
	High	97.41 (19.23)	
Subjective health status	Unhealthy	80.32 (17.65)	4.68 (< 0.001)
	Healthy	96.68 (17.04)	
Smoking	Yes	89.79 (20.70)	0.99 (0.325)
	No	94.71 (17.81)	
Alcohol	Yes	94.52 (17.54)	0.11 (0.915)
	No	94.25 (18.44)	
Body mass index	Underweight	93.76 (17.08)	0.78 (0.505)
	Normal	95.39 (18.21)	
	Overweight	88.63 (14.60)	
	Obese	94.57 (20.87)	

M: mean, SD: standard deviation

### Correlations among major study parameters

Table 4 shows the relationships between major variables such as age, GPA, BMI, SJL, sleep duration, light exposure, depression symptoms, and QOL. QOL was statistically significantly negatively correlated with age, SJL, and depression symptoms. In addition, there was a borderline positive correlation between QOL and GPA.

**Table 4:** Correlation between variables (*N* = 198)

	Age	GPA	BMI	Chronotype	Social jetlag	Sleep duration	Light exposure	Depression symptoms	QOL
	*r*(*p*)	*r*(*p*)			*r*(*p*)	*r*(*p*)	*r*(*p*)	*r*(*p*)	*r*(*p*)
Age	1								
GPA	0.131 (0.065)	1							
BMI	0.05 (0.487)	− 0.14 (0.043)	1						
Chronotype^a^	0.19 (0.008)	0.05 (0.525)	− 0.02 (0.764)	1					
Social jetlag	− 0.07 (0.348)	− 0.07 (0.360)	− 0.11 (0.123)	− 0.23 (0.001)	1				
Sleep duration	− 0.07 (0.318)	0.02 (0.809)	0.01 (0.845)	0.17 (0.014)	− 0.16 (0.022)	1			
Light exposure	0.14 (0.046)	0.11 (0.128)	0.02 (0.837)	0.09 (0.220)	− 0.01 (0.857)	0.10 (0.148)	1		
Depression symptoms	− 0.07 (0.298)	− 0.23 (0.001)	0.06 (0.383)	− 0.13 (0.080)	0.34 (< 0.001)	− 0.09 (0.214)	− 0.05 (0.517)	1	
QOL	− 0.17 (0.017)	0.14 (0.050)	− 0.05 (0.454)	0.11 (0.111)	− 0.31 (< 0.001)	0.06 (0.377)	0.08 (0.275)	− 0.40 (< 0.001)	1

GPA: grade point average, BMI: body mass index, QOL: quality of life.

^a^Total MEQ-K scores.

### Predictors of OQL

As a result of the post-hoc Harman’s single factor test, the total variance extracted by one factor was 23.11% less than the threshold value of 50%; hence, common method bias could be ruled out. Multiple regression analyses were performed to identify the predictors of QOL. Variables that statistically significantly differed or were correlated with QOL at *p* < .10 (age, subjective health status, GPA, chronotype, SJL, and depression symptoms) were added to the model using the entered method.

First, there was no multicollinearity among the independent variables, and a variance inflation factor ranging from 1.07 to 1.21 was confirmed. Further, independence among the error terms was confirmed, with a Durbin-Watson index of 2.05 (du = 1.83, 4-du = 2.17), satisfying the assumptions for regression analysis. This regression model explained 27.8% of the variance in QOL. We tested the normality of residuals through the Kolmogorov-Smirnov test (*Z* = 0.05, *p* = .788) and homoscedasticity using the Breusch-Pagan test (*χ*^*2*^ = 3.64, *p* = .726). The statistically significant predictors of QOL were age (*β* = .-19, *p* = .003), subjective health status (*β* = 0.21, *p* = .001), SJL (*β* = .-17, *p* = .013), and depression (*β* = .-33, *p* < .001). Depression symptoms was the most potent predictor variable (Table 5).

**Table 5:** Factors influencing quality of life (*N* = 198)

Variables	Quality of life						95% CI	
	*B*	*SE*	*β*	*t*	*p*	VIF	Lower	Upper
(Constant)	116.75	13.31		8.78	< 0.001		90.51	143.17
Age	-0.95	0.32	− 0.19	3.02	0.003	1.09	-1.57	-0.33
Subjective health status^a^	10.77	3.26	0.21	3.27	0.001	1.09	4.22	17.06
Grade point average	2.82	2.61	0.07	1.08	0.281	1.07	-2.33	7.99
Chronotype^b^	0.10	0.16	0.04	0.60	0.551	1.12	-0.22	0.42
Social jetlag	-0.69	0.27	− 0.17	2.51	0.013	1.19	-1.23	-0.15
Depression symptoms	-0.79	0.16	− 0.33	4.95	< 0.001	1.21	-1.11	-0.48
	Adjusted *R*^*2*^ = 0.278, *F* = 13.66 (*p* < .001)							
Breusch-Pagan test (*χ*^*2*^ = 3.64, *p* = .726), Kolmogorov-Smirnov test (*Z* = 0.05, *p* = .788)								

SE: standard error, VIF: variance inflation factor, CI: confidence interval

^a^Dummy variable (reference): Subjective health status (unhealthy)

^b^Total MEQ-K scores

## Discussion

In this study, we performed multiple regression analyses with major study parameters, including SJL, to identify the predictors of nursing students’ QOL during the COVID-19 pandemic. The results identified age, subjective health status, SJL, and depression symptoms as the predictors of nursing students’ QOL, with depression symptoms being the most potent predictor. This is similar to the results of a study conducted with nursing students prior to the COVID-19 pandemic [39], in which SJL, positive emotional state, and depressive symptoms were identified as the predictors of QOL. This also coincides with the results of a study that analyzed the predictors of stress in health professional students during the COVID-19 pandemic [9], where age, self-rated health, the presence of sleep problems, life satisfaction, and the use of coping strategies were identified as significant predictors of HRQOL.

In this study, depression symptoms was the most powerful predictor variable of QOL in nursing students. Although the inverse relationship between depression and QOL is well known [55, 56], it is possible that limitations of activity owing to the COVID-19 pandemic and concerns over academic performance and long-term employment [13] further intensified nursing students’ depression, rendering it the most potent predictor of QOL. In previous studies, resilience, having online experience, and being well prepared for online learning significantly predicted nursing students’ QOL [31]. Taken together, supporting nursing students to adjust to the changes in the educational system owing to the COVID-19 pandemic and enhancing their resilience to cope with an unstable situation could help reduce depression symptoms and improve nursing students’ QOL.

In this study, the mean SJL and sleep duration were 1 h and 19 min and 7 h and 9 min, respectively. Compared to the mean SJL of 1 h and 36 min and mean sleep duration of 6 h and 30 min in a study on nursing students in South Korea prior to the COVID-19 pandemic, the mean SJL decreased while the mean sleep duration increased in our study, showing positive changes. This is similar to previous findings regarding the COVID-19 pandemic increasing students’ sleep duration and positively changing their sleep habits [32]. Studies reported that delaying school start times extends students’ sleep duration, which has a positive effect on adolescents’ biological sleep phase [32, 57–59]. In the present study, increased sleep duration and reduced SJL compared to that before school closures from COVID-19 could be interpreted in the same context. Seemingly, the shift toward online learning owing to the COVID-19 pandemic increased students’ sleep duration, as they no longer needed to wake up early in the morning for practicum, and their SJL had decreased by taking online courses at their preferred times.

A study conducted with adolescents who began homeschooling owing to the COVID-19 lockdown in Switzerland showed that students’ weekday sleep duration increased by 75 min and their HRQOL improved. Longer sleep duration was significantly associated with improved HRQOL [32]. Furthermore, students’ depressive symptoms were significantly inversely associated with HRQOL [32]. We also observed in this study that students’ sleep duration increased and their SJL decreased, which could potentially improve their QOL. However, considering findings showing that an increased prevalence of depression among college students was strongly associated with poor sleep quality [60], and that high sleep quality has a significant emotional impact on students and thus could alleviate mental health problems [61], further studies are needed to examine the effects of sleep quality and duration on nursing students’ health and QOL. Moreover, students’ depression symptoms had a negative impact on HRQOL even after adjusting for the positive effects of increased sleep duration during the COVID-19 pandemic [32], which warrants a closer analysis of COVID-19-related depression symptoms in college students.

In a study on the changes of SJL owing to the COVID-19 pandemic, 46% of the participants showed decreased SJL; however, there were also participants who had no changes in SJL, based on which the said authors suggested that a shift to a later chronotype, as opposed to changes in the SJL itself, plays a key role [62]. Our study population was relatively young, with a mean age of 26.41 years, and although their SJL did decrease compared to the pre-COVID-19 period, it was still large. Hence, considering that SJL has a significant effect on depression symptoms [38, 39], it is possible that depression symptoms increased owing to young age, late chronotype, and high SJL.

Subjective health status was also identified as a predictor of QOL; that is, QOL improved with better subjective health status. In a previous study, students’ chronic absenteeism had a negative impact on their health, and a sense of academic achievement was linked to a higher level of general health [63]. During the COVID-19 pandemic, students who participated in more hours of online classes demonstrated a higher physical HRQOL, and frustration and stress symptoms caused by study disruption impaired students’ physical HRQOL [20]. In our study, students who effectively adjusted to online learning and thus had less stress from participating in new classes had better subjective health status, which could have had a positive impact on QOL. Furthermore, good friend networks and support boosts subjective health status [64], and greater family and friend support improves physical HRQOL by increasing physical activity [20, 65]. Thus, we cannot eliminate the possibility that students who maintained good relationships with others and received adequate support from their social networks during the COVID-19 lockdown had better subjective health status and thus high QOL.

In this study, age was a significant predictor of QOL, with QOL declining with advancing age. In contrast, a study that conducted cluster analyses of a college student sample in Brazil reported that students in the low QOL group tended to be younger than their higher QOL counterparts [66]. Moreover, although gender was not a significant predictor in our study, Azzi et al. [66] reported that the low QOL group was predominantly women, requiring further examination of the associations between age, gender, and QOL. However, female students tend to be more vulnerable to psychological distress than male students [67], and data show that external support helps female students cope with stressful life events and lower stress, anxiety, and depression levels [68]. Hence, more elaborate approaches tailored to age- and gender-specific features are needed to help college students in maintaining mental health and improving QOL.

Positive thinking, resilience, and exercise lower severe mental health impacts [69]; thus, the current results should be comprehensively reviewed to develop measures to protect college students’ mental health and improve their QOL during the COVID-19 pandemic. In addition, a randomized clinical trial investigating the effects of sleep training on college students’ mental health and QOL reported that mental health and QOL improved after three months, as opposed to immediately after training [70]. Consequently, a long-term approach should be adopted when planning sleep interventions that aim to reduce SJL and improve sleep quality in college students.

This study has some limitations. First, we used data from a self-report questionnaire to measure the major study parameters; thus, the findings are vulnerable to recall bias. While we used validated instruments to measure chronotype and SJL, studies should utilize objective measures to assess participants’ circadian parameters and sleep-related characteristics. Second, this study employed a cross-sectional design; thus, the causal relationships among major variables cannot be established. A longitudinal study is needed to examine the predictors of QOL in nursing students, with a focus on SJL. Third, this was a quantitative study; therefore, we relied on a structured questionnaire. Future qualitative studies that provide an in-depth understanding of the features of nursing students’ QOL and their predictors during the COVID-19 pandemic are needed. Fourth, we attempted to include participants from diverse backgrounds by using an open call online survey. Hence, the impact of institutional differences was not controlled for. In subsequent studies, by limiting the sample to participants from a few target institutions, multilevel modeling can be considered and school condition should be investigated in detail (i.e., timetable scheduling, credits obligation, majors) to understand the impact of SJL. The generalizability of the findings of this study is limited given that the sample was not representative of the entire nursing student population. Thus, replication studies with larger study samples need to be conducted.

Despite these limitations, this study is significant in that it paid attention to nursing students’ SJL, which could be altered by lifestyle changes during the COVID-19 pandemic, and we analyzed the predictors of QOL during the COVID-19 pandemic. The results may be useful as evidence when developing measures to boost nursing students’ QOL in new living and educational environments brought upon by the pandemic.

## Conclusion

This study identified the predictors of nursing students’ QOL during the COVID-19 pandemic. These predictors have largely remained consistent compared to the pre-COVID-19 period, and SJL, subjective health status, and depression symptoms were still significant predictors of QOL in nursing students. While we could not investigate the direct causes of depression symptoms in this study, we suspect that inadequate interactions with peers or other students at school, anxiety over academic performance owing to sudden changes in the educational system, and clinical practicum difficulties during the COVID-19 pandemic are linked to nursing students’ depression. As subjective health status is a significant predictor, it is necessary to help students maintain good physical and mental health and perceive their health status positively during the pandemic. Furthermore, considering that positive emotional state, resilience, and coping strategies were previously identified as significant predictors of QOL, it is vital to provide continued attention and proper education to foster positive coping strategies and resilience against various adversities caused by the pandemic to help students overcome depression symptoms.

## Data Availability

The data sets used and/or analyzed during the current study are available from the corresponding author on reasonable request.

## List of abbreviations

BMI: Body Mass Index
CES-D: Center for Epidemiological Studies Depression Scale
COVID-19: Coronavirus Disease 2019
GPA: Grade Point Average
HRQOL: Health-Related Quality of Life
MCTQ: Munich Chronotype Questionnaire
MEQ-K: Korean Version of the Morningness-Eveningness Questionnaire
QOL: Quality of Life
SJL: Social Jetlag
